# Feasibility of Metatranscriptome Analysis from Infant Gut Microbiota: Adaptation to Solid Foods Results in Increased Activity of Firmicutes at Six Months

**DOI:** 10.1155/2017/9547063

**Published:** 2017-08-24

**Authors:** Floor Hugenholtz, Jarmo Ritari, Lotta Nylund, Mark Davids, Reetta Satokari, Willem M. de Vos

**Affiliations:** ^1^Laboratory of Microbiology, Wageningen University, Dreijenplein 10, 6703 HB Wageningen, Netherlands; ^2^Department of Basic Veterinary Medicine, University of Helsinki, Helsinki, Finland; ^3^Functional Foods Forum, University of Turku, Turku, Finland; ^4^Laboratory of Systems and Synthetic Biology, Wageningen University, Dreijenplein 10, 6703 HB Wageningen, Netherlands; ^5^RPU Immunobiology, Department of Bacteriology and Immunology, University of Helsinki, Helsinki, Finland

## Abstract

Newborns are rapidly colonized by microbes and their intestinal tracts contain highly dynamic and rapidly developing microbial communities in the first months of life. In this study, we describe the feasibility of isolating mRNA from rapidly processed faecal samples and applying deep RNA-Seq analysis to provide insight into the active contributors of the microbial community in early life. Specific attention is given to the impact of removing rRNA from the mRNA on the phylogenetic and transcriptional profiling and its analysis depth. A breastfed baby was followed in the first six months of life during adaptation to solid food, dairy products, and formula. It was found that, in the weaning period, the total transcriptional activity of Actinobacteria, mainly represented by* Bifidobacterium*, decreased while that of Firmicutes increased over time. Moreover, Firmicutes and Actinobacteria, including the canonical Bifidobacteria as well as* Collinsella*, were found to be important contributors to carbohydrate fermentation and vitamin biosynthesis in the infant intestine. Finally, the expression of* Lactobacillus rhamnosus*-like genes was detected, likely following transfer from the mother who consumed* L. rhamnosus* GG. The study indicates that metatranscriptome analysis of the infant gut microbiota is feasible on infant stool samples and can be used to provide insight into the core activities of the developing community.

## 1. Background

After birth, newborns are rapidly colonized by intestinal microbiota originating from the surrounding environment, including maternal faecal microbes and vaginal or skin species, depending on the mode of delivery [1, 2]. It is generally assumed that the first colonizers of the infant intestine are facultative anaerobes, such as* Streptococcus*,* Enterococcus* and* Lactobacillus* spp., and* Escherichia coli* [3, 4], followed by anaerobic bacteria such as* Bifidobacterium, Clostridium*, and* Ruminococcus* spp. [5, 6]. Defining a normal intestinal microbiota at early age is challenging, since the composition and temporal patterns of the microbial communities vary widely among infants. During maturation the variation among infants becomes smaller when obtaining a more adult-like microbiota composition, but this has recently been found to take over 5 years of time [7–9].* Bifidobacterium* and to a lesser extent* Bacteroides* are considered the predominant bacterial genera colonizing the early infant gut when the infant is breastfed as these bacteria have the rather unique capacity to degrade human milk oligosaccharides [10]. Introduction of solid foods significantly alters the gut microbiota, switching the microbial composition to be more dominated by* Bacteroides* and* Clostridium* spp. [7, 9, 11]. The increase in abundance of the phyla Firmicutes and Bacteroidetes after weaning is an indication of the adaptation to a more complex diet [6, 7].

Various culturing approaches have shown that there is vertical transmission of specific bacteria from mother to baby. This was shown elegantly for endogenous Bifidobacteria [12] as well as probiotic bacteria consumed by the mother that in the case of* Lactobacillus rhamnosus* GG were found in the baby [13, 14]. Culture-independent evidence for maternal transmission of early life microbiota is limited, notably as the baby microbiota differs so much from that of the mother. In some cases signatures of parental microbes have been described to be present in the infant microbiome [15] and recently it was reported that the microbiota in children at 3 years of age shows signatures present similar to their own mother and were not seen in between children and unrelated mothers [16].

While considerable attention has been given to the compositional development during early life, only recently high throughput omics-based approaches have been applied. A recent study analysed the faecal metagenome during the first year of life and observed striking differences between vaginal and C-section delivered infants [7]. Moreover, some early studies addressed the transcriptome of Bifidobacteria in the intestinal tract of babies that were breastfed or on a formula-diet and showed differential expression of genes involved in sugar catabolism, exopolysaccharide production, or folate biosynthesis [17]. Moreover metatranscriptome analysis in infants and their mothers showed differences in expression of higher capacity of mucin utilization, higher capacity folate biosynthesis, and decreased starch degradation in infants [18]. Global metatranscriptome analysis in the GI tract microbiota could enable the elucidation of the specific functional roles microbes have in this complex community. Initial metatranscriptome studies in the human large intestine revealed that different functions are expressed between individuals while core functions of the microbiota appeared to be consistently expressed among individuals [19–21]. These findings imply that metatranscriptomics could provide insight into the differential activity profiles in the gut microbiota, enabling the reconstruction of the metabolic activity profile of microbial communities.

In this study we describe the feasibility of using deep RNA-Seq analysis to get further insight in the active contributors of the microbial community in early life. Specific attention is given to the rapid sampling and impact of removing rRNA from the mRNA on the phylogenetic and transcriptional profiling and its analysis depth. A breastfed baby was followed in the first six months of life during adaptation to solid food, dairy products, and formula. The results indicate that* Bifidobacterium* is an active member of the community and over time various members of the Firmicutes become more active that are involved in vitamin production and sugar metabolism at 6 months.

## 2. Methods

### 2.1. Subject, Dietary Information, and Sampling

One vaginally born, breastfed, Finnish baby girl was followed during the introduction of first solid foods into the diet. Faecal samples were collected at three time points, specifically at the ages of 131, 165, and 171 days. These samples were given with consent of the mother and ethics are within national and international regulation. The time points and number of samples were taken for practical reasons as the faecal samples were taken at home and immediately processed in RNAlater as to preserve the mRNA as good as possible. The infant did not receive any probiotic supplementation, but her mother consumed dairy products containing* Lactobacillus rhamnosus* GG. Both infant and her mother were healthy and did not receive any antibiotics during the study period. At the first time point (131 days), the infant consumed exclusively breast-milk and some mashed potato and roots as the first baby food, which was extended in the second time point (165 days) to fruits, vegetables, and grain and meat products. Before the last time point the infant was also introduced to dairy products and formula milk (Table 1).

### 2.2. RNA Extraction, rRNA Removal, cDNA Synthesis, Library Preparation, and Illumina MiSeq 2500 Sequencing

Fresh faecal samples were collected and immediately processed as described previously [22]. For this purpose, one ml RNAlater was added to each gram of sample and stored at −70°C until later processing. The amount of faecal material used for the RNA extraction was 6.81 g for T1, 4.12 g for T2, and 4.42 g for T3, respectively. Total RNA was extracted as described before using the Macaloid procedure [23] and final RNA concentrations were measured with the NanoDrop 1000. Here we obtained 35.09, 38.04, and 31.82 *μ*g of total RNA for T1, T2, and T3, respectively. From sample T2, 5 *µ*g of RNA was also used for rRNA removal by using the Ribo-Zero kit (Epicentre), which is a magnetic beads-based rRNA hybridization technique to remove the 23S, 16S, and 5S rRNA from the sample. One sample was taken only, to see the effect of the rRNA removal in the sequencing results. This portion was further named T2_M, while the sample containing total RNA was labelled T2_T. 500 ng of total RNA from the samples T1, T2_T, and T3 and due to the rRNA removal 244 ng of mRNA of T2_M was used for cDNA synthesis and library construction (TrueSeq RNA Sample Preparation Kit, Illumina, San Diego, CA). Due to the quality differences, slightly different lengths were taken for the sequencing: T1: 300–700 bp, T2_T: 300–570 bp, T2_M: 300–500 bp, and T3: 300–500 bp. Sequencing was performed using the Illumina MiSeq instrument on 2 × 150 bp paired end mode. Between 3 and 4 million reads were obtained per sample.

### 2.3. RNA-Seq Data Processing

The raw reads data are available in the MG-RAST server under the following accession codes: 4621794.3, 4621795.3, 4621796.3, 4621797.3, 4621798.3, 4621799.3, 4621800.3, and 4621801.3. After fastq quality filtering reads were taxonomically assigned by matching against a human intestinal 16S rRNA database (https://github.com/microbiome/HITdb, Ritari et al. unpublished) at 97% identity threshold. For the mRNA analysis rRNA reads were rapidly filtered from the samples using SortmeRNA (version 1.2), after which the remaining reads were assembled using idba_ud using the pipeline described previously [24]. Essential single copy genes (ESCG), identified using HMM search (http://hmmer.org/), were taxonomically classified using MEGAN after being aligned against full NR database. The remainder of the proteins was taxonomically classified by aligning them against all proteins in the NR database belonging to members of nine identified bacterial orders. Functional annotation was performed to all predicted protein sequences by assignment of KEGG orthology identifiers using the KEGG KAAS server. Expression levels of the predicted ORFs were determined by aligning the reads against the assembly using bowtie2 and counting reads mapped to each ORF using BEDtools.

## 3. Results and Discussion

### 3.1. *In Vitro* rRNA Removal Increases Functional Transcription Depth

**Figure 1:**
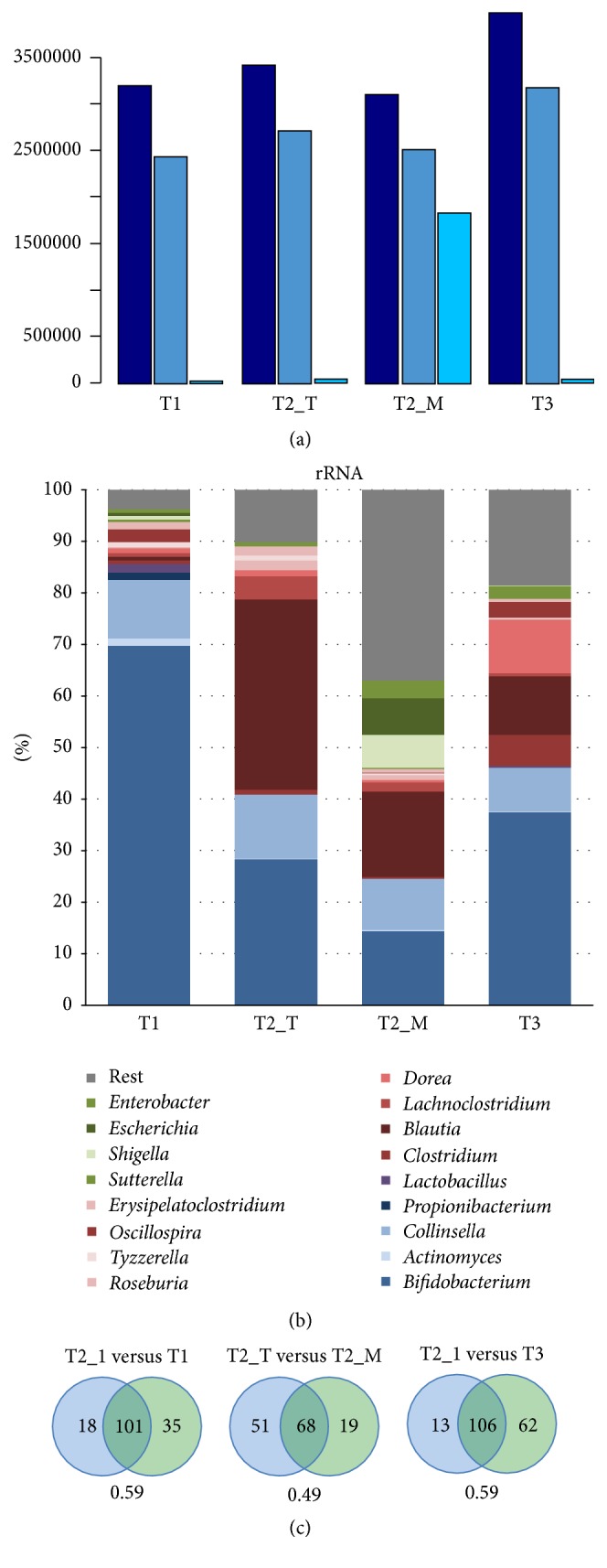
(a) Numbers of reads at different stages of in silico filtering. Raw reads (dark blue), after quality filtering (medium blue) and after quality and rRNA filtering (light blue). (b) Taxonomic profiles based on 16S rRNA. (c) Numbers of shared genera between T2_T and other samples. The Jaccard index below the Venn diagrams shows the relationship between shared and all genera.

Since rRNA is known to dominate the composition of total bacterial RNA, we employed an* in vitro* rRNA removal procedure to evaluate its effect on the content and accuracy of the sequenced RNA pool. Thus from one of the samples (T2_M), rRNA was removed prior to the RNA-Seq analysis to enrich for mRNA, while in all other samples total RNA was sequenced. Thus, it allowed the comparison of the same sample with and without rRNA removal (i.e., T2_T and T2_M). After quality filtering, a total number of approximately 2.5 million reads were obtained in all samples (Figure 1(a)). However, when analysing these, it was observed that reads derived from rRNA were dominating in samples where* in vitro* rRNA removal was not applied; in silico filtering of rRNA sequences by means of matching to 5S, 16S, and 23S rRNA gene databases removed about 99% of reads, with the exception of sample T2_M where only about 28% of reads were removed. This showed that T2_M contained much less rRNA than untreated samples and confirmed the efficiency of the* in vitro* rRNA removal procedure. As a consequence, for sample T2_M, the number of filtered mRNA reads was approximately 250-fold higher than that of the untreated samples.

### 3.2. Impact of rRNA Removal on 16S rRNA Based Taxonomic Profiles

Comparison of taxonomical profiles of samples with and without* in vitro* rRNA removal (T2_T versus T2_M) showed a significant difference between the samples (Figure 1(b)). Indeed, the difference between T2_T and T2_M was more pronounced than the difference between samples T2_T and T3, representing different time points. The difference was especially evident in the abundance of* Bifidobacterium *spp. that showed an approximately twofold reduction in sample T2_M as compared to the identical but not treated sample T2_T (Figure 1(b)). Furthermore, the amount of 16S rRNA sequences of* Blautia *was also approximately 2-fold reduced, while that of Proteobacteria increased over 15-fold in T2_M, altogether indicating a strong effect of the rRNA removal by the kit. In addition, the number of common genera between the samples T2_T and T2_M was lower than that between other samples; T2_T had more in common with T1 and T3 than with T2_M (Figure 1(c)). Moreover, comparing the log relative abundances between T2_T and T2_M indicated that there were several noncorrelating genera, which were abundant in T2_M but rare in T2_T (Supplementary Figure 1 in Supplementary Material available online at https://doi.org/10.1155/2017/9547063).

Altogether, the results indicate that the used* in vitro* rRNA removal procedure causes bias to the taxonomic composition, most likely because the procedure does not target all taxonomic groups equally. Thus it generates taxonomic profiles with different bacterial content and abundances when compared to untreated samples. This has also been observed for other rRNA removal methods in a study with a synthetic community consisting of 5 genera that are not common inhabitants of the intestinal tract. [25]. Here, we confirm and extend this analysis and show that the rRNA removal using the Ribo-Zero kit (Epicentre) specifically reduces the rRNA fraction of the Firmicutes and Actinobacteria from the intestinal microbiome of the infant, while increasing that of the Proteobacteria. Hence, taxonomic analysis based on 16S rRNA sequences from* in vitro* treated material should be taken with caution, even though there would be enough reads left after the removal.

### 3.3. Metatranscriptome

For further analysis at mRNA-level, the data was filtered to remove rRNA sequences, adapter sequences, and poor quality reads. SortMeRNA [26] was used to rapidly filter out rRNA sequences using the precompiled databases for eukaryotes, bacteria, and archaea. To determine the function and taxonomy of the mRNA reads, they were merged and de novo assembled into larger contigs, creating a single contig reference set for all samples. A total of 11558 contigs larger than 300 bp could be assembled with an overall length of 9,843,629 bases. These contigs encoded a total of 16,422 predicted open reading frames. Altogether 30 to 68% of the mRNA reads could be mapped and assigned to protein-encoding gene regions (Table 2).

**Figure 2:**
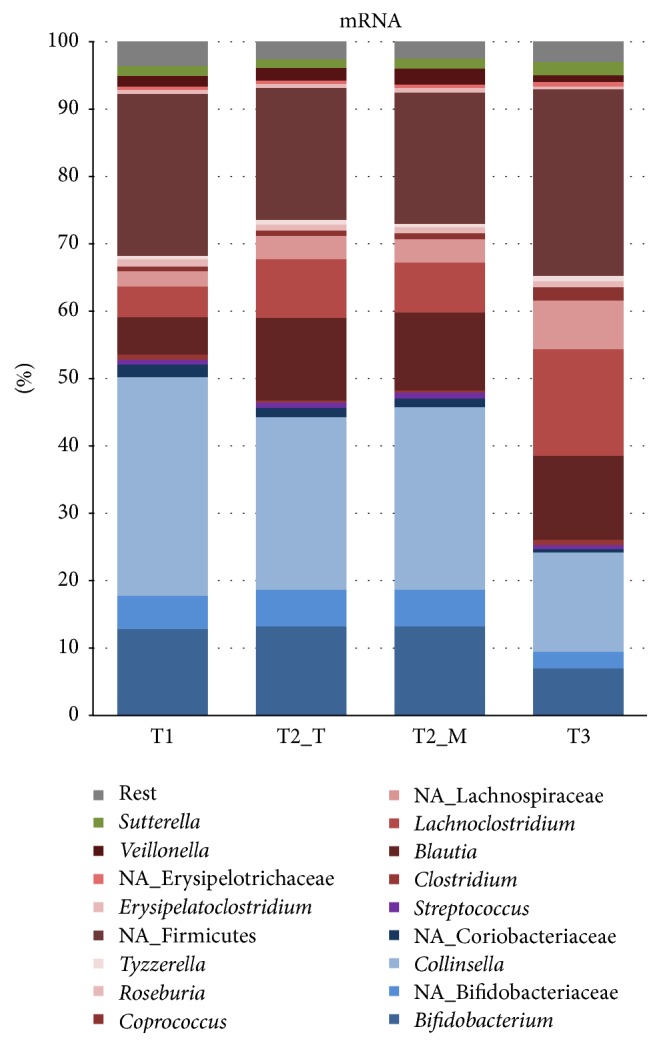
Taxonomic profiles based on mRNA.

The protein assigned mRNA reads were used for a detailed taxonomic analysis (Figure 2). As the mapping is not without bias and quite variable (Table 2), we observed various differences in the phylogenetic composition of the baby samples based the 16S rRNA (Figure 2) and mRNA data (Figure 2). These differences in abundances may also be explained by varying gene expression levels that do not necessarily reflect the real abundances of underlying bacterial groups. Remarkably, the phylogenetic composition, based on the mapped mRNA, appeared to be not affected by the* in vitro* rRNA removal as indicated by a Pearson correlation coefficient of T2_T and T2_M samples of 0.99 (Supplementary Figure 2). This suggests that the rRNA removal is likely to preserve mRNA levels with high fidelity.

**Figure 3:**
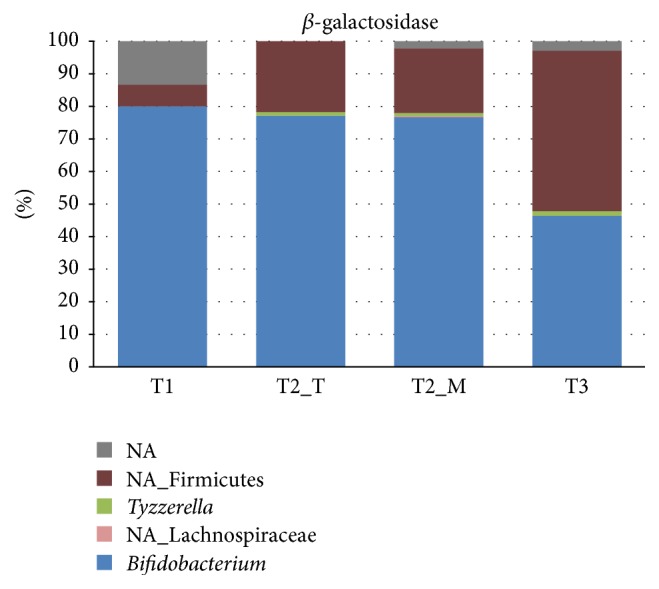
Taxonomic profiles based on expression of the *β*-galactosidase gene.

The main taxonomic groups expressing protein-encoding transcripts belonged to* Bifidobacterium, Collinsella*,* Blautia, Lachnoclostridium*, and unidentified Firmicutes (Figure 2). The expression of mRNA derived from* Bifidobacterium* and* Collinsella* decreased from T1 to T3, most likely due to the introduction of new solid foods into the infant diet after T1. As the weaning process proceeds, a wider variety of carbon sources becomes available in the infant intestine, thus supporting the establishment of more diverse microbial community. This was demonstrated by the high expression of bifidobacterial *β*-galactosidase while the infant received exclusively breast-milk (T1), whereas unidentified Firmicutes were the main producer of this enzyme at T3 when the infant diet included also a variety of solid foods and dairy products (Figure 3). Transcripts derived from Bacteroidetes were hardly found in the dataset. While Bacteroidetes have been reported to be present during weaning [6, 7], their absence in 3-month healthy and breastfed babies is not unusual [18, 27]. As the rRNA-derived composition also indicated a low level of Bacteroidetes (Figure 1(b)), this indicated that the infant's gut microbiota during weaning is still adapting towards more adult-like composition. However we must point out here that we have not obtained specific information on the sampling time during the day and the exact amount and frequency of the food intake; this could change activity patterns considerably and should be taken into consideration in future experiments.

### 3.4. In-Depth Functional and Taxonomic Distributions of Metabolic Pathways

**Figure 4:**
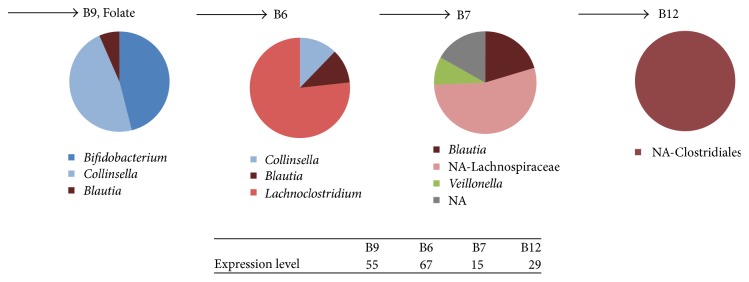
Relative taxonomic profiles based on the final enzyme for the corresponding vitamin metabolism of sample T2_M. KEGG numbers for the final step in the corresponding vitamin pathways for this figure are K00287 (B9, Folate), K00868 (B6); K01012 (B7, Biotin); K02233 (B12).

Metagenome analyses have indicated that the production of vitamins may be one of the benefits of the early life intestinal microbiota [7, 9]. However, evidence based on expression studies is lacking apart from indications of folate biosynthesis by Bifidobacteria in 8-month-old infants [17]. The sequencing depth in sample T2_M was sufficient for determining the taxonomic distribution of the vitamin pathways (Supplementary Figure 3) as well as that of sugar transport and the taxonomic distribution of the glycosylases expression. Remarkably, the expressed genes involved in biosynthesis of the vitamin folate (B9) reflect similar taxonomic distribution as the *β*-galactosidase and the overall mRNA expression profiles to some extent, with* Bifidobacterium, Collinsella*, and* Blautia* as the main contributors to folate metabolism (Figure 4). Other vitamins such as B6, B7, and B12 were each produced by quite different organisms, including* Blautia, Lachnoclostridium*, and Lachnospiraceae, which mainly belonged to phylum Firmicutes. This indicates that other bacteria than the canonical* Bifidobacterium* spp. contribute to important functions such as vitamin production at 6 months of age.

**Figure 5:**
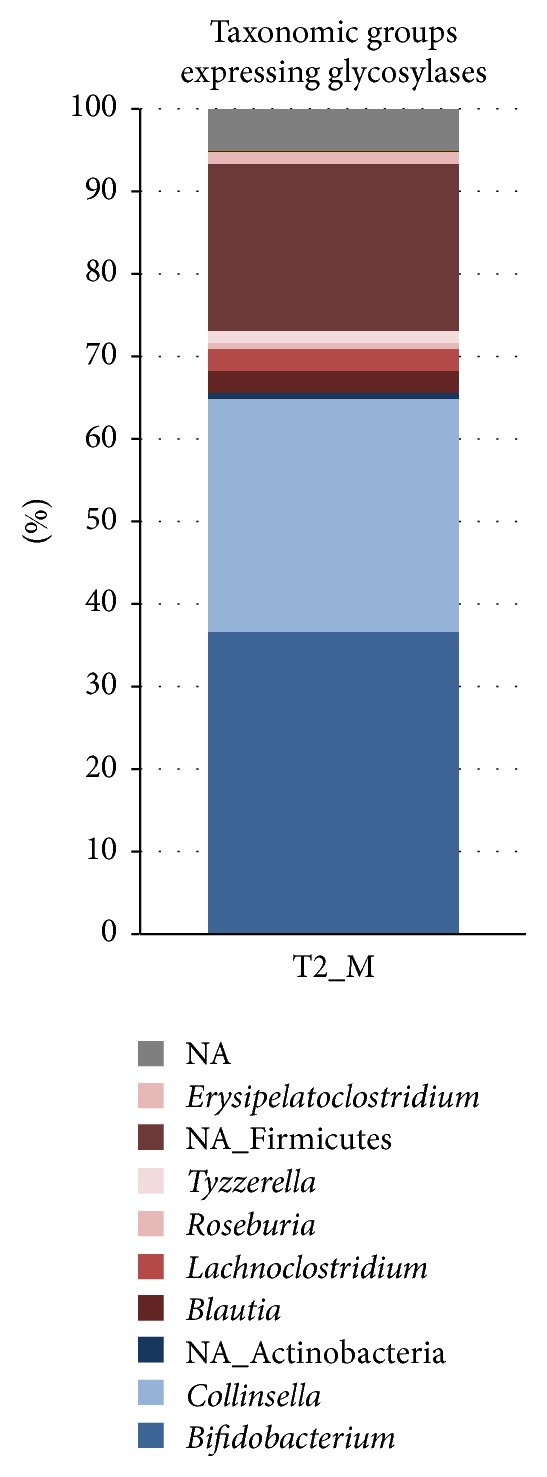
Taxonomic profiles based on expression of the glycosylases genes in sample T2_M.

We were also able to detect in sample T2_M the expression of a large variety of genes for sugar transporters belonging to PEP-dependent phosphotransferase (PTS) or ATP binding cassette (ABC) systems that are characterized by the involvement of multiple proteins (Table 3). Not all transcripts of the transporter module proteins functions were equally expressed but this may be due to differential gene expression or transcript stability. Metagenomic analysis had also revealed the presence of these transporters in infants of 4 and/or 12 months [7]. Altogether, the expression of these transporter genes indicates that a large range of sugars is transported by the gut microbiota in an infant of approximately 6 months old. Moreover, the expression of glycosidase genes specific for the hydrolysis of the transported saccharides was also detected, indicating the functional use of these sugars, including alpha- and beta-glycosidases (altogether denoted as glycosylase genes) (Figure 5; Supplementary Table 1). These glycosidase genes had been previously found to be present before the introduction of solid food [6]. While Actinobacteria expressed the largest amount of sugar transport and degradation genes, around 25% of the transcripts derived from Firmicutes, indicating that members of both phyla are important contributors to the carbohydrate degradation.

### 3.5. Expression Levels of Genes from* Lactobacillus rhamnosus* GG

**Figure 6:**
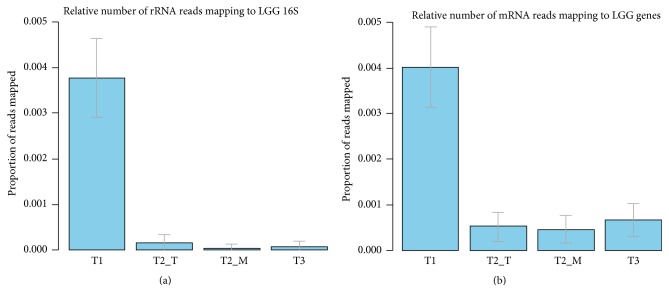
Relative numbers of mRNA (b) and rRNA (a) read mapping to LGG. Error bars represent standard deviations based on binomial sampling.

While breastfeeding, the mother consumed dairy products containing* Lactobacillus rhamnosus *GG (LGG), which is very common in many dairy products and drinks in Finland and has the capacity to strongly interact with human mucus via its protruding pili [28]. Previously, it has been shown that mother-infant transfer of LGG may occur frequently [14]. To study whether we could deduce the presence of LGG or related bacteria in the transcriptome of the studied infant, mRNA reads were mapped to LGG genome at 95% similarity threshold. This analysis confirmed the expression of LGG-like genes, including these present in variable regions present in LGG but absent in many other* L. rhamnosus* strains [29]. Comparative analysis revealed significantly more LGG-like genes (% of total mRNA reads) in sample T1 when compared to samples T2 and T3 (Figure 6). The same result was seen when mapping rRNA reads from faecal samples to LGG 16S rRNA sequence at 97% similarity. While only a few different LGG-like functions could be detected in the transcriptome of sample T1, in that of sample T2_M, the expression of the LGG-like genes related to glycolysis is clearly visible, indicative of its high activity (Supplementary Figure 4). Since the sample T1 represents the microbiota of an infant with breast-milk as the main source of nutrients and whose mother consumed LGG containing products, it may be speculated that LGG was acquired during or soon after the birth from the mother. At later time points the abundance of LGG-like transcripts dropped, suggesting that LGG would then not permanently colonize the infant intestinal tract ecosystem. However, as this coincides with the introduction of formula and solid food, one may speculate that these factors also contribute to the clearance of LGG.

## 4. Conclusions

We demonstrate here the feasibility of metatranscriptome analysis using high throughput RNA-Seq on the infant gut to get in-depth insight in the active community structure in the weaning period. A relevant technical observation is that rRNA removal with Ribo-Zero kit (Epicentre) has a pronounced effect on the rRNA distribution but not on that of mRNA. It was found that in the weaning period of the breastfed infant a number of major activity changes occurred with a decrease in activity of the Actinobacteria, mainly represented by* Bifidobacterium*, and an increase in activity of members of the Firmicutes increased in activity over time. Moreover, the Firmicutes and Actinobacteria, including the canonical Bifidobacteria and Collinsella, were found to be important contributors to vitamin production in the infant intestine, including that of vitamin B6, B7, B9, and B12. Finally, the mother consumed the probiotic LGG and we detected LGG-like transcripts in the infant transcriptome, indicating that LGG has been transferred, is an active member at the earliest time point, and is decreasing in abundance and activity during weaning, showing that LGG is not likely to remain a permanent member of the intestinal microbiota. The here described approach and pipeline allow for further longitudinal analysis of infant activity to complement infant cohort metagenome studies that are presently developing.

## Supplementary Material

Supplementary Figure 1: Log relative abundances of common genera between 2D_1 and 2D_2.
Supplementary Figure 2: Correlations of the expression pattern between the samples.
Supplementary Figure 3: Overall expression of mRNA functions on the visualization tool iPATH2.0.
Supplementary Figure 4: Overall expression of mRNA functions from LGG on the visualization tool iPATH2.0.

## Figures and Tables

**Table 1:** Overview of the infant diet.

Sample code	T1	T2	T3
Age (days)	131	165	171
Breast milk	Exclusively	Yes	Yes
Formula	No	No	Yes
Potato & roots	First baby food	Yes	Yes
Fruits & berries	No	Yes	Yes
Vegetables	No	Yes	Yes
Grain products	No	Yes	Yes
Meat/chicken/fish/eggs	No	Yes	Yes
Milk & dairy products	No	No	Yes

**Table 2:** Sequencing, assembly, and mapping information of the samples.

	Total reads (pairs)	mRNA reads (pairs)	Mapped (single)	% mapped
T1	1591215	6229	3801	30.5
T2_T	1710009	13749	17570	63.9
T2_M	1550435	1084270	1480261	68.3
T3	1986769	15661	15657	50.0

**Table:** (a) PTS transporter modules

Module number	*p* value	Adjusted *p* value	Number of proteins in modules	Number of transcribed proteins-encoding genes	Description
M00266	0	0.006	3	2	Maltose and glucose-specific II component
M00269	0	0.006	3	2	Sucrose-specific II component
M00268	0	0.006	3	2	Arbutin-like II component
M00273	0	0.006	3	2	Fructose-specific II component
M00270	0.001	0.011	4	2	Trehalose-specific II component
M00277	0.001	0.011	4	2	N-Acetylgalactosamine-specific II component
M00267	0.031	0.555	3	1	N-Acetylglucosamine-specific II component
M00282	0.031	0.555	3	1	D-Glucosamine-specific II component
M00272	0.031	0.555	3	1	Arbutin-, cellobiose-, and salicin-specific II component
M00303	0.031	0.555	3	1	N-Acetylmuramic acid-specific II component
M00279	0.031	0.555	3	1	Galactitol-specific II component
M00283	0.031	0.555	3	1	Ascorbate-specific II component

**Table:** (b) ABC sugar transporter modules

Module number	*p* value	Adjusted *p* value	Number of proteins in modules	Number of transcribed protein-encoding genes	Description
M00601	0	0.009	3	2	Putative chitobiose transport system
M00219	0.001	0.018	4	2	AI-2 transport system
M00211	0.025	0.48	2	1	Putative ABC transport system
M00217	0.038	0.716	3	1	D-Allose transport system
M00210	0.038	0.716	3	1	Putative ABC transport system
M00605	0.05	0.949	4	1	Glucose/mannose transport system
M00206	0.05	0.949	4	1	Cellobiose transport system
M00606	0.05	0.949	4	1	N,N′-Diacetylchitobiose transport system
M00197	0.05	0.949	4	1	Putative fructooligosaccharide transport system
