# Supplementary material for: Feasibility of Metatranscriptome Analysis from Infant Gut Microbiota: Adaptation to Solid Foods Results in Increased Activity of Firmicutes at Six Months
Source: Int J Microbiol. 2017 Aug 24;2017:9547063. doi: 10.1155/2017/9547063 (PMC5587937; doi:10.1155/2017/9547063)

Table and Figure Legends

Supplementary Figure 1. Log relative abundances of common genera between 2D_1 and 2D_2.

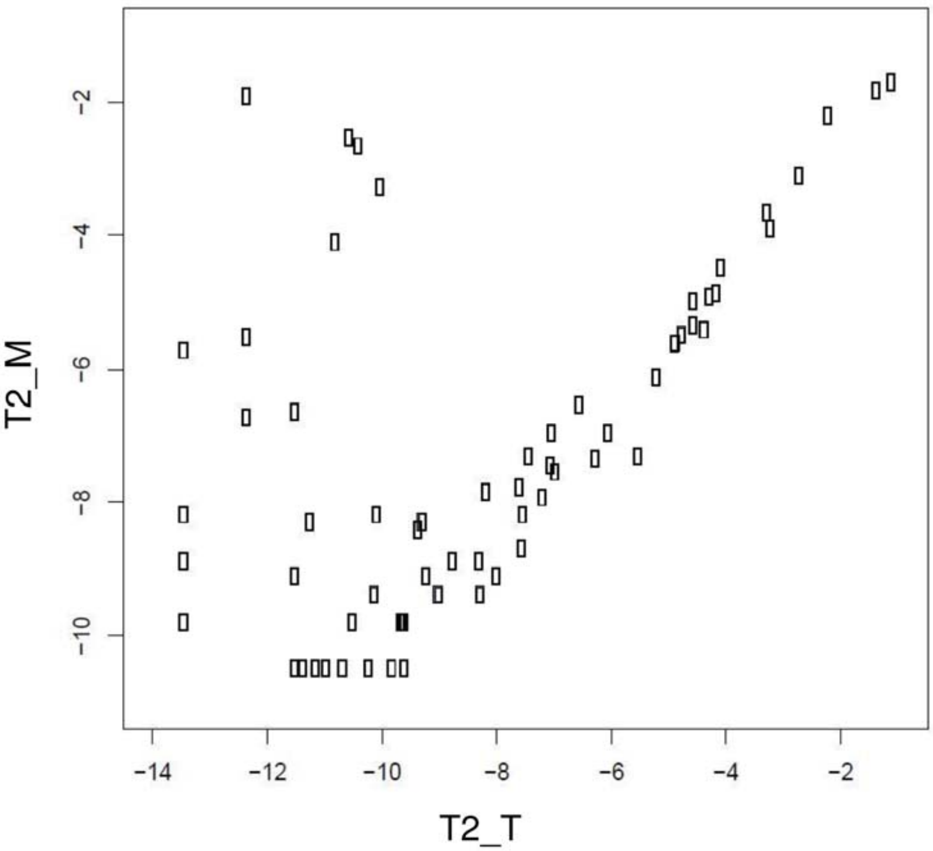

Supplementary Figure 2. Correlations of the expression pattern between the samples.

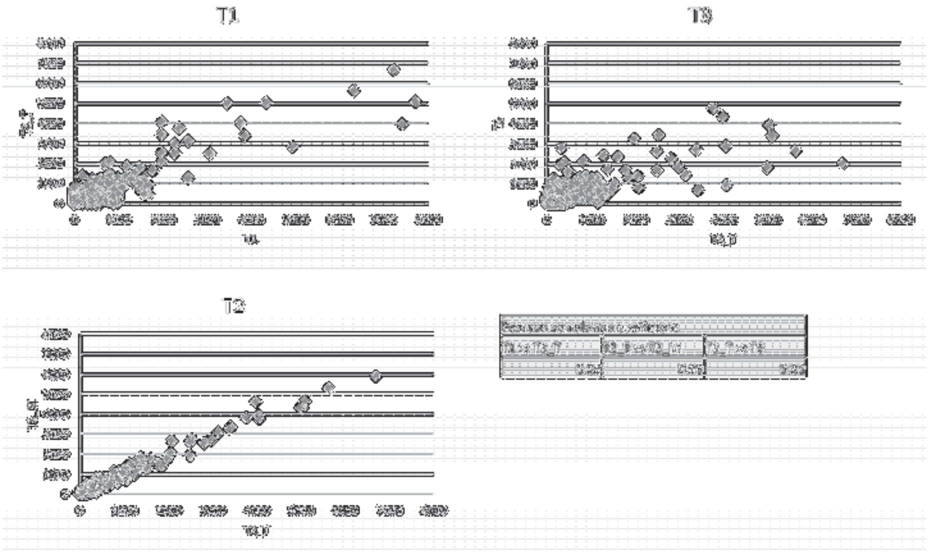

Supplementary Figure 3. Overall expression of mRNA functions on the visualization tool iPATH2.0.

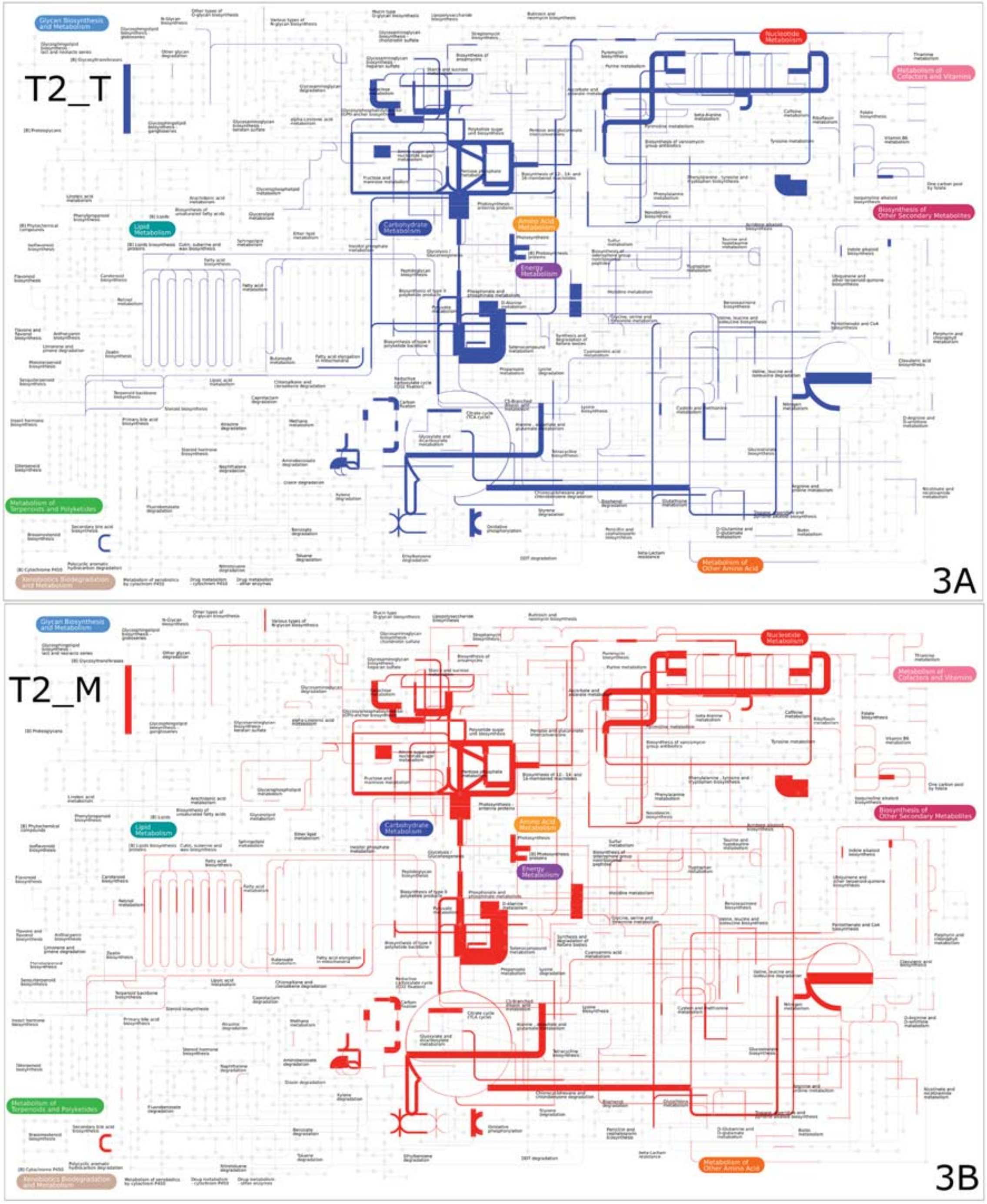

Supplementary Table 1. List of identified glycosylases in sample T2_M

K01176	amyA, malS; alpha-amylase;
K01182	oligo-1,6-glucosidase;
K01186	NEU1; sialidase-1;
K01187	malZ; alpha-glucosidase;
K01190	lacZ; beta-galactosidase;
K01191	alpha-mannosidase;
K01192	MANBA, manB; beta-mannosidase;
K01193	sacA; beta-fructofuranosidase;
K01197	hya; hyaluronoglucosaminidase;
K01198	xynB; xylan 1,4-beta-xylosidase;
K01200	pullulanase;
K01205	NAGLU; alpha-N-acetylglucosaminidase;
K01206	FUCA; alpha-L-fucosidase;
K01207	nagZ; beta-N-acetylhexosaminidase;
K01209	abfA; alpha-N-arabinofuranosidase;
K01210	glucan 1,3-beta-glucosidase;
K01215	dexB; glucan 1,6-alpha-glucosidase;
K01220	lacG; 6-phospho-beta-galactosidase;
K01223	bglA; 6-phospho-beta-glucosidase;
K01227	mannosyl-glycoprotein endo-beta-N-acetylglucosaminidase;
K01232	glvA; maltose-6'-phosphate glucosidase;
K01234	nplT; neopullulanase;
K01239	iunH; purine nucleosidase;
K01240	URH1; uridine nucleosidase;
K01243	mtnN, mtn, pfs; adenosylhomocysteine nucleosidase;
K01246	tag; DNA-3-methyladenine glycosylase I;
K01250	rihA; pyrimidine-specific ribonucleoside hydrolase;
K01811	yicI; alpha-D-xyloside xylohydrolase;
K02438	treX, glgX; glycogen operon protein;
K03332	fruA; fructan beta-fructosidase;
K03575	mutY; A/G-specific adenine glycosylase;
K03648	UNG, UDG; uracil-DNA glycosylase;
K03660	OGG1; N-glycosylase/DNA lyase;
K05349	bglX; beta-glucosidase;
K05520	pfpI; protease I;
K05546	GANAB; alpha 1,3-glucosidase;
K06113	abnA_B; arabinan endo-1,5-alpha-L-arabinosidase;
K07407	galA, rafA; alpha-galactosidase;
K08309	slt; soluble lytic murein transglycosylase;
K12111	ebgA; evolved beta-galactosidase subunit alpha;
K12308	bgaB, lacA; beta-galactosidase;
K12309	GLB1, ELNR1; beta-galactosidase;
K12373	HEXA_B; hexosaminidase;
K15532	yteR, yesR; unsaturated rhamnogalacturonyl hydrolase;

K15719	NCOAT, MGEA5; protein O-GlcNAcase / histone acetyltransferase
K15921	xynD; arabinoxylan arabinofuranohydrolase;
K15923	AXY8, FUC95A, afcA; alpha-L-fucosidase 2

Supplementary Figure 4. Overall expression of mRNA functions from LGG on the visualization tool iPATH2.0.

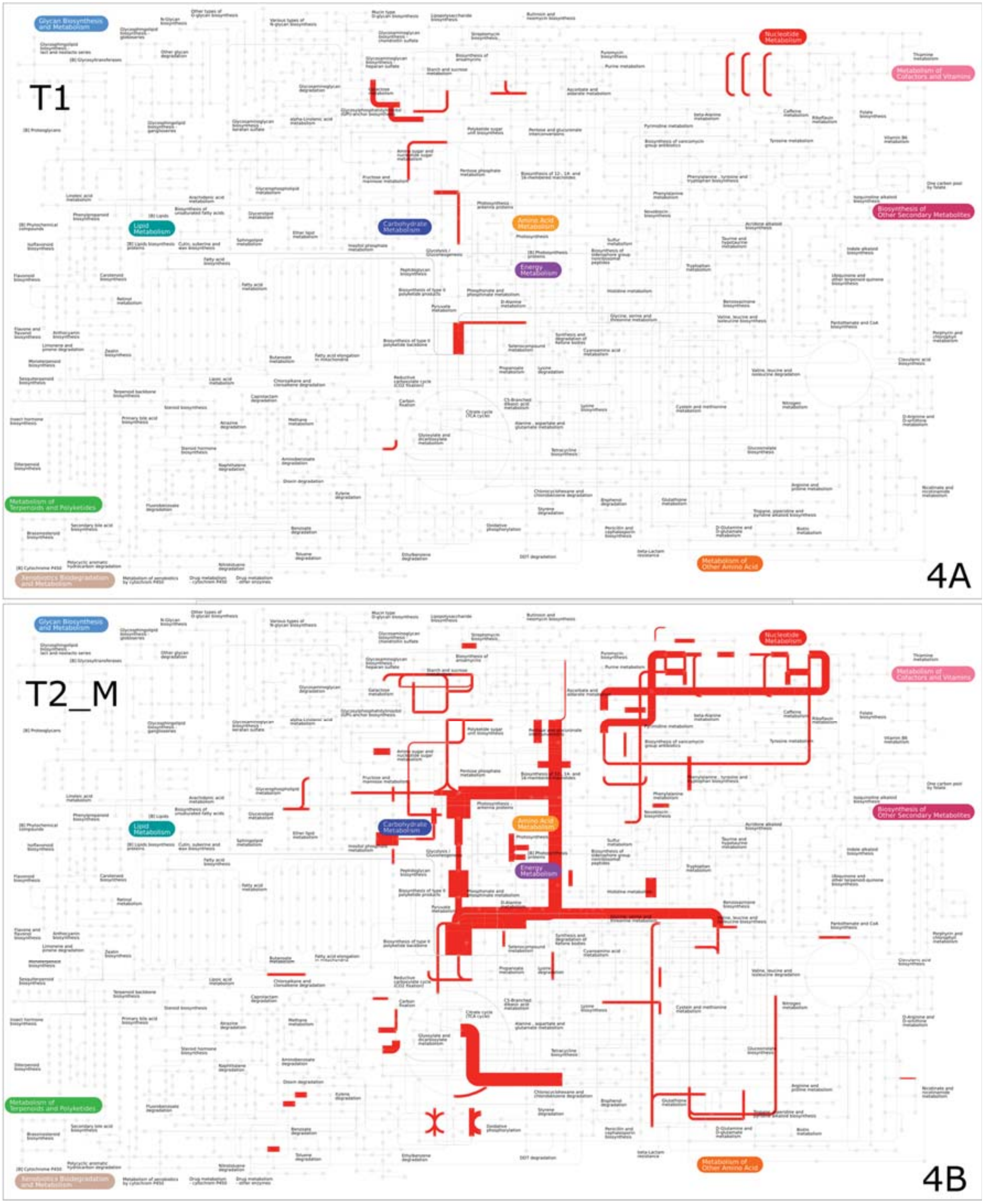

Supplement: Supplementary file 1 — Supplementary Figure 1: Log relative abundances of common genera between 2D_1 and 2D_2. Supplementary Figure 2: Correlations of the expression pattern between the samples. Supplementary Figure 3: Overall expression of mRNA functions on the visualization tool iPATH2.0. Supplementary Figure 4: Overall expression of mRNA functions from LGG on the visualization tool iPATH2.0. [file 9547063.f1.pdf]
